# Improving the homogeneity of diffraction based colours by fabricating periodic patterns with gradient spatial period using Direct Laser Interference Patterning

**DOI:** 10.1038/s41598-019-44212-4

**Published:** 2019-05-24

**Authors:** Bogdan Voisiat, Wei Wang, Max Holzhey, Andrés Fabián Lasagni

**Affiliations:** 1 Institut für Fertigungstechnik, Technische Universität Dresden, George-Baehr-Str. 3c, 01069 Dresden, Germany; 2 Fraunhofer-Institut für Werkstoff- und Strahltechnik IWS, Winterbergstr. 28, 01277 Dresden, Germany

**Keywords:** Laser material processing, Surface patterning, Imaging techniques

## Abstract

This study focuses on the development of a strategy to produce periodic structures with a variable spatial period for increasing the homogeneity of structural colours by means of direct laser interference patterning. Using a four-beam interference configuration, hole-like periodic arrays are produced on stainless steel with a 70 ps pulsed laser source operating at 532 nm laser wavelength. The laser processing parameters are optimised for obtaining patterns with the highest possible diffraction efficiency and thus showing the highest possible colour intensity. A model for calculating the required spatial period to obtain a defined colour under specific conditions of illumination and observation angles is presented. A very good agreement between the captured structural colour spectrum and the real visible spectrum of light was obtained. In addition, a strategy for mixing holographic colours, in particular for obtaining the white colour is developed. Finally, the developed model is successfully integrated into machine software, in order to automatically process images that exhibit required colours at certain viewing conditions. The produced patterns are characterised using confocal microscopy and the efficiency of the first diffraction order was measured by optical spectroscopy.

## Introduction

Laser surface texturing is an effective method to produce microstructures on different materials in a single-step process for a large range of applications^1–3^. In the last decades, different technologies such as laser induced periodic surface structures (LIPSSs) and Direct Laser Interference Patterning (DLIP) have been utilised to produce repetitive patterns on the surfaces of various materials (including metals, semiconductors and dielectrics) in order to functionalise these surfaces^4–8^.

In particular, when repetitive periodic patterns are fabricated for instance on metallic surfaces, characteristic colour effects (e.g. rainbow colour) have been reported, making this principle very interesting for producing variable information marking elements with high potential in marking industry^9–11^.

LIPSS are a universal phenomenon that occurs on different solids when they are irradiated with polarised laser radiation^12^. These structures are in general characterised by (quasi-) periodic lines, whose spatial period correlates with the wavelength of the used laser source and whose orientation depends on the polarisation of the radiation^4^. Furthermore, different mechanisms have been suggested for explaining the formation of these features, such as interference effects of the incident laser with surface plasmon waves and self-reorganization of the irradiated material^13,14^. LIPSS have been observed on a large variety of materials (including metals, semiconductors and dielectrics)^15^ using a broad range of pulse durations, ranging from continuous wave irradiation^16^ down to a few femtoseconds (fs)^17^.

One of the most promising applications of LIPSS arises from the fact that they can act as diffractive gratings, and thus are capable to generate structural colours. Depending on the size of the LIPSS (spatial period) as well as their orientation, different shades of structural colours throughout the visible part of the spectrum are can be achieved. For instance, using a fs-laser, the optical properties of different metals, including aluminium, gold and platinum, have been modified using this concept^4,18^. Other examples of LIPSS for inducing structural colour on steels, copper and semiconductors have been also reported^19–23^.

A very relevant research in this field has been reported by Dusser *et al*.^20^. This pioneer work showed the possibility of achieving well-defined structural colour by a precise control of size and orientation of LIPSS by changing different laser parameters such as pulse energy, polarization direction, spot size and overlapping of spots. A drawback of using LIPSS for the generation of structural colours is the imperfect final surface topography, which reduces the diffraction efficiency and thus the diffracted light intensity. In addition, since LIPSS present in general a line-like geometry, the diffracted colours are visible mainly in the perpendicular direction of the periodic ripples.

This drawback can be overcome using Direct Laser Interference Patterning (DLIP), which is based on the formation of interference patterns by overlapping two or more coherent laser beams. The interference patterns consist on a periodic modulation of the laser intensity, which can be used to selectively modify the surface of a material^24–28^. Furthermore, numerical simulations have permitted to determine the most relevant variables that can be used to control the shape and size of the patterns, including laser light polarization, number of beams, relative intensity and incidence angle^29,30^. In contrast to LIPSS, the spatial period can be controlled independently of the target material by changing the angles of interception between the beams or by switching the laser wavelength. However, since the laser wavelength in general cannot be easily modified, the spatial period is controlled by adjusting the interfering angle. For example using a two-beam setup, line-like geometries can be produced with a spatial period (Λ) defined by Eq. 1:

1 $$\Lambda =\frac{\lambda }{2\,\sin (\theta )},$$

where *λ* is the laser wavelength and *θ* is the half angle between the two incident beams. As it can be observed from Eq. 1, the minimum achievable spatial period is half of the used laser wavelength.

Using this method, the fabrication of periodic patterns for obtaining structural colours has been reported as well^31^. The main idea behind this work is to produce spots (40–80 µm in diameter) containing the interference patterns, which are used to produce the periodic gratings locally on the surface of the material. By varying the spatial period of the produced holographic-spots, different colours can be obtained at different positions under identical illumination conditions. Furthermore, this technology has been already used for producing decorative elements on large size metallic sleeves (300 mm in diameter and 300 mm in length) for R2R-hot embossing applications^32^. For the largest decorative elements (117 mm × 68 mm) a total of 553 million of individual pixels (spots) were fabricated with 3 different spatial periods. In addition, DLIP optical modules have been also developed permitting a fully automatised variation of the spatial period and geometry^33,34^.

In recent years, various commercially available DLIP systems have been developed, such as DLIP-µFAB and DLIP-Cube, where the DLIP optics have been combined with galvanometer scanning systems allowing a significant increase in throughput^35^.

As mentioned before, both methods (LIPSS and DLIP) have been employed to locally control the morphology of produced patterns and thus to obtain structural colours. However, due to the nature of the diffraction phenomena and especially for large areas, periodic patterns under real illumination conditions exhibit rainbow colours (the whole spectrum of the incident light) making it difficult to produce decorative elements with a homogenous structural colour. In addition, white colour cannot be produced using this approach, since it results from a combination of different wavelengths (corresponding to red, green and blue colours).

This study focuses on the development of a strategy to produce periodic structures with variable spatial period for increasing the homogeneity of structural colours using the DLIP method. By using a four-beam interference optics and a ps-pulsed laser source, symmetric hole-like periodic arrays are produced on stainless steel. Firstly, the laser processing parameters are optimised for obtaining patterns with the highest possible diffraction efficiency (and thus with the highest colour intensity). A theoretical model to calculate the required spatial period for obtaining a defined colour under specific conditions of illumination and observation is presented. In addition, a strategy for producing white colour is introduced. Finally, as an example a stainless steel substrate is structured using the strategies presented in this work to reproduce an arbitrary image with different colours.

## Materials and Methods

### Sample preparation

Flat samples of stainless steel (304) were utilised for the DLIP laser process since this material can be used in a wide range of applications, in particular where decorative elements can be applied. The used samples had a thickness of 0.7 mm with an average surface roughness (*Ra*) of 52 nm. The substrates were used as received.

### Picosecond direct laser interference patterning

The laser processing was performed using a self-developed DLIP system (DLIP-μFAB, from Fraunhofer IWS). Four laser beams were used to obtain hole-like interference patterns. The area of the interference ranged from 30 μm to 120 µm allowing the formation of decorative motifs with a resolution between 200 and 850 DPI. The laser beam radius was measured at a level of 1/e^2^ using a beam profiling camera. The compact DLIP system is equipped with a Nd:YAG laser source (Q-switched, from NeoLase), which operates at 532 nm laser wavelength, emitting the fundamental transverse mode (TEM00) with a M² (laser beam quality factor) below 1.2. The laser source provides 70 ps pulses with a repetition rate fixed to 1 kHz, yielding pulse energies up to 26 µJ. The main component of the DLIP system consists of a DLIP optical head in which the input laser beam is split into four sub-beams by a diffractive optical element (DOE). By using a fused silica prism, these sub-beams are parallelised and finally overlapped using a convex lens. Since small spatial periods are required for obtaining the decorative elements, a lens with a short focal distance (60 mm) was used. This optical setup allows the automatic control of the spatial period from 1.75 µm to 4.59 µm, which is achieved by changing the angle of incidence of the beams at the samples’ surface (additional information about this setup has been already published elsewhere^36^). The samples were moved in the horizontal (X) and vertical (Y) directions using translational stages (Aerotech, USA) at a speed of 100 mm/s. These stages permit to treat areas up to 200 mm × 200 mm. All experiments were carried out in ambient environment and no post treatment was performed.

### Surface characterization

The treated surfaces were characterised using Confocal Microscopy (LeicaSCAN DCM3D) with a 150X magnification objective providing lateral and vertical resolutions of 140 nm and 1 nm, respectively. Using this objective, a total area of 351 µm × 264 µm could be recorded in each measurement. Optical photographs were acquired with a high-resolution camera (Nikon D5100) equipped with zoom objective (AF-S DX NIKKOR 18–105 mm 1:3.5–5.6 G ED VR) and fixed under controlled position and inclination with respect to the treated samples using a standard tripod.

## Results and Discussion

### Theoretical calculations

It is well known that when light interacts with a periodic structure, it is diffracted with a certain diffraction angle, which depends on the wavelength as well as on the spatial period of the repetitive structure. This angle can be calculated for the first diffraction order, using Eq. 2 (for light reaching the surface perpendicularly):

2 $${\theta }_{1storder}=\arcsin (\lambda /\Lambda ),$$

where Λ is the spatial period of the periodic structure and λ the wavelength of light diffracted at the angle θ_1st order_.

**Figure 1:**
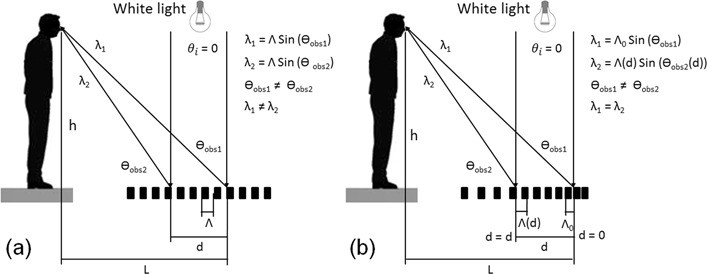
Schematic representation of diffraction of white light by a surface containing (**a**) a periodic structure with a constant spatial period Λ and (**b**) a gradient periodic surface structure with a variable period following Eqs 3 to 5.

However, a characteristic “rainbow” colour will be perceived especially for large areas, since the light reaching the observer (from the first diffraction orders) has different viewing angles (e.g. θ_obs1_, θ_obs2_) and thus different wavelengths (λ_1_, λ_2_ see Fig. 1a).

Differently, if a variable structure is produced, which means that the spatial period changes depending on the position at the substrate, it is possible to calculate the needed “local” spatial period in order to see only a specific colour depending on the angle of incidence of the light source and the position of the observer. This situation can be obtained by applying Eqs 3 to 5 and is illustrated in Fig. 1b:

3 $${\theta }_{{\rm{o}}{\rm{b}}{\rm{s}}1}=\arctan (\frac{L}{h}),$$

4 $$\Lambda (d)=\frac{m\,\lambda }{\sin ({\theta }_{i})-\,\sin ({\theta }_{{\rm{o}}{\rm{b}}{\rm{s}}2}\,(d))},$$

5 $${\theta }_{{\rm{o}}{\rm{b}}{\rm{s}}2}(d)=\arctan (\frac{L-d}{h}),$$

where L and h describe a lateral and vertical position of the observer, respectively, m corresponds to a difraction order, d is a relative position of the spatial period, θ_i_ is an angle of incident of the light source (in Fig. 1b, θ_i_ = 0) and $\Lambda$ (d) is the required spatial period as a function of the position d.

Due to the characteristic of the DLIP method, a precise control of the spatial period as a function of the position d is possible, which might be very difficult to achieve using other laser based methods (e.g. LIPSS). This strategy was applied in the next subsections for increasing the homogeneity of structural colours using the DLIP method.

### Improving the efficiency of 1^st^ diffraction orders

**Figure 2:**
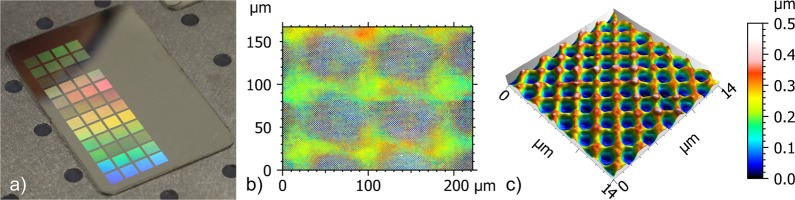
(**a)** Photograph of the stainless steel surface treated with different number of pulses (from 1 to 5) and laser fluences (from 0.7 to 11.4 J/cm²); (**b**) confocal image of DLIP holographic pixels on the steel surface with a diameter of approximately 50 µm; (**c**) typical hole-like pattern with a spatial period of 1.8 µm and a structure depth of 0.3 µm (the used laser fluence was 1.9 J/cm² and 3 pulses were applied).

In a first approach, the influence of the structure depth of the produced hole-like patterns on the efficiency of the diffracted light was evaluated for different spatial periods. An example of a treated stainless steel sample, using the four-beam interference setup is shown in Fig. 2a. In this case, the spatial period was 1.8 µm and the patterns were obtained with different spot sizes (50, 80, 95 and 120 µm) and laser pulse energies (9, 15, 19 and 26 µJ) that correspond to the fluence ranging between 0.7 and 11.4 J/cm². Also the number of laser pulses applied per spot was varied between 1 and 5 pulses. As it can be seen in Fig. 2c, the material was selectively ablated at the interference maxima positions obtaining a characteristic hole-like geometry. The total treated area for an individual pixel can be also seen in Fig. 2b, corresponding to a diameter of approximately 50 µm.

**Figure 3:**
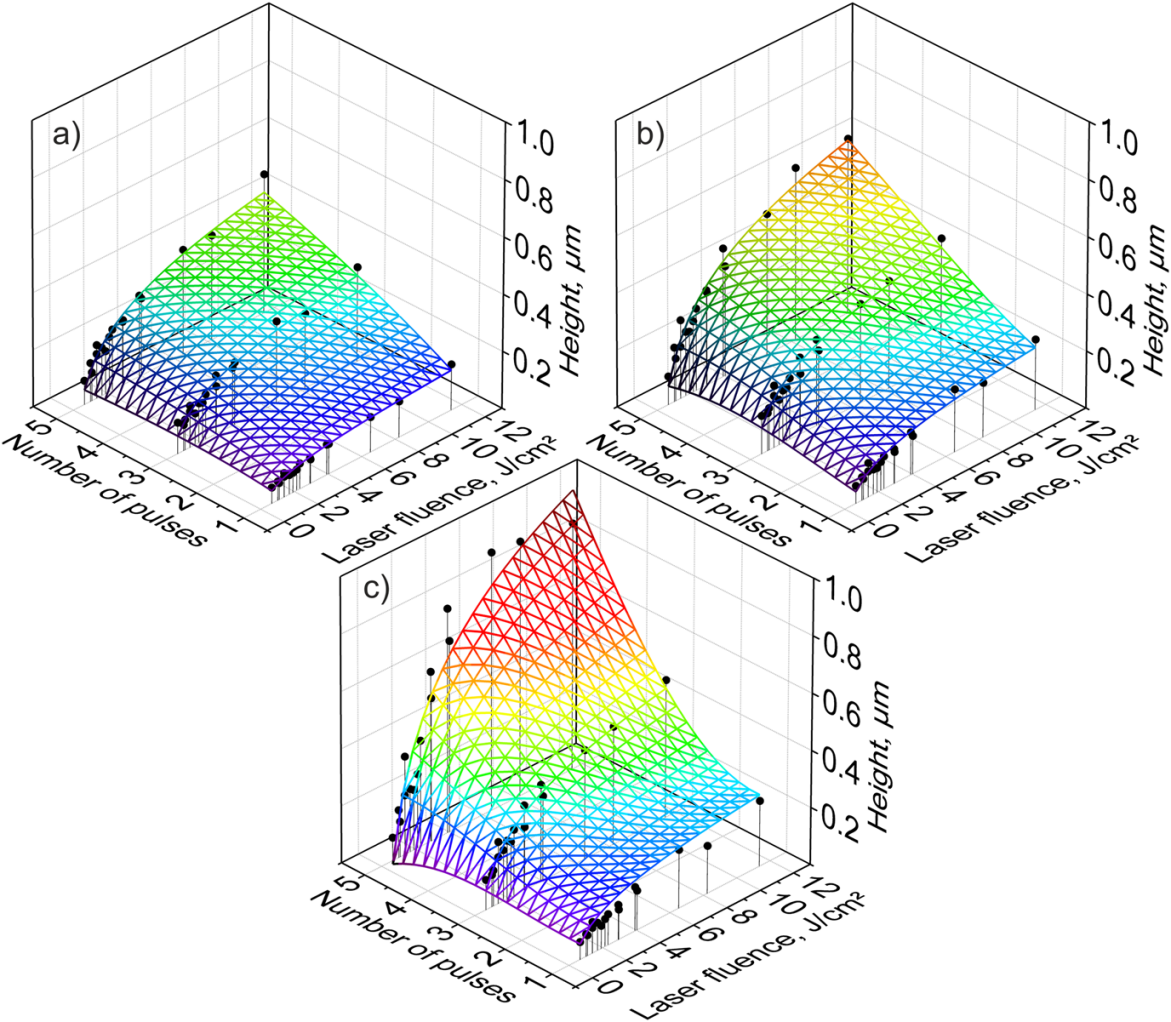
Depth of the produced hole-like patterns as a function of the applied number of laser pulses and laser fluence for (**a**) 5.7 µm, (**b**) 2.6 µm and (**c**) 1.8 µm spatial periods.

To control the structure depth of the produced hole-like patterns, both the number of laser pulses and the laser fluence were changed. The influence of the above mentioned parameters on the structure depth is shown in Fig. 3 for three different spatial periods: 1.8 µm, 2.6 µm, 5.7 µm.

As it can be seen, byincreasing the number of used pulses or the laser fluence, the structure depth could be increased as well up to 0.40 µm, 0.52 µm and 0.79 µm, for the spatial periods 1.8 µm, 2.6 µm and 5.7 µm, respectively.

**Figure 4:**
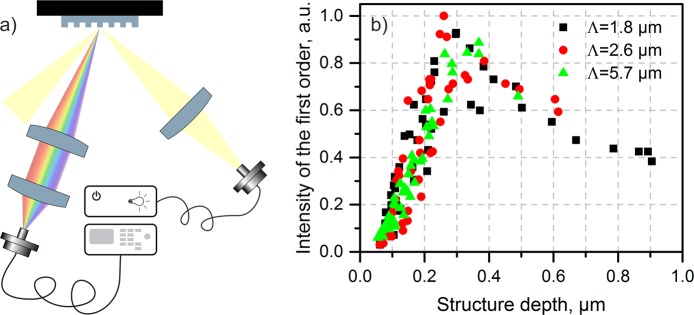
(**a**) Setup of the spectroscopic device used to measure the intensity of the first order diffraction efficiency and (**b**) measured first diffraction order intensity for the hole-like periodic patterns with 1.8 µm, 2.6 µm and 5.7 µm spatial periods. The maximal measured intensity was obtained for a structure depth of approximately 0.3 µm for all periods.

In order to determine the relation between the structural colour intensity and the fabricated periodic structure depth, quantitative measurements of the diffraction efficiency for the first diffraction orders were performed using the setup shown in Fig. 4a. The measured diffraction intensities for the evaluated spatial periods (1.8 µm, 2.6 µm and 5.7 µm) are shown in Fig. 4b. As it can be seen, the maximum efficiency was obtained for a structure depth of approximately 0.3 µm for all spatial periods. For structure depths over and below this value, lower diffraction efficiencies were measured for all the studied periods. In consequence, for the next experiments we targeted a structure depth of ~0.3 µm for all utilised spatial periods in order to obtain the highest colour intensity.

The intensity of the diffraction orders is directly related to the gratings’ diffraction efficiency, which according to scalar diffraction theory depends on the arbitrary groove shape within the gratings’ period^37^. For example, the highest first order efficiency of the most popular rectangular shaped grating in reflection mode is reached when the groove depth is equal to one quarter of the incident wavelength. The grooves of the laser fabricated gratings are not square-shaped, therefore the optimum depth is different but still related to the incident wavelength. In this case, the peak represents the average optimum depth for all wavelengths within the analysed visible spectrum.

**Figure 5:**
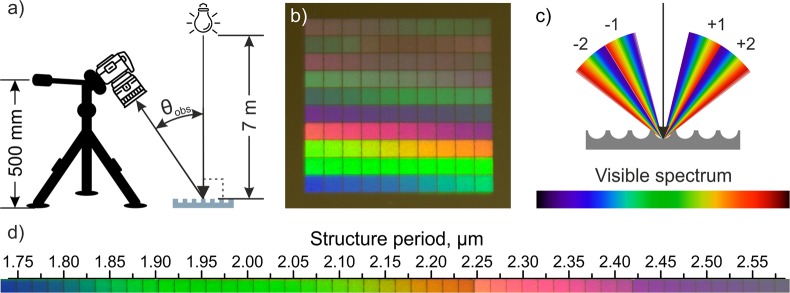
(**a**) Schematic representation of the used sample and observer (camera) positions; (**b**) photograph of a treated stainless steel sample with spatial periods ranging from 1.75 µm to 4.59 µm (size of each square is 1.5 mm); (**c**) schematic representation of diffracted withe light for the first and second diffraction orders; (**d**) optical micrograph of the observed structural colours depending on the spatial period for an observation angle of 22°.

In a second set of experiments, the stainless steel surfaces were processed with different spatial periods ranging from 1.75 µm to 4.59 µm. Then structured areas were photographed at the same conditions using the photo camera fixed at a permanent position on a tripod as shown in Fig. 5a. The camera was positioned at a lateral position distance L of 205 mm and at height h of 500 mm, resulting in an observation angle υ_obs_ of 22.3°. The results show that for this condition, the visible spectrum of light could be seen for spatial periods ranging from 1.74 µm to 2.59 µm (the 5 bottom rows in Fig. 5b, which are plotted in respect to the structure period in Fig. 5d), corresponding to wavelengths between 467 nm and 694 nm (calculated using Eq. (2)). It is known that the visible spectrum ranges from 380 nm (violet colour) to 750 nm (red colour) (see Fig. 5c) corresponding to grating periods from 1.35 µm to 2.78 µm, when the observation conditions of the 1^st^ diffraction order are the same as in Fig. 5a. It means that the spectrum that was captured with the camera is not covering the violet part of the 1^st^ diffraction order’s spectrum. Instead, this part of the spectrum was visible on the right side of the captured colour spectrum (between 2.4 µm and 2.6 µm periods), which corresponds to the blue side of the 2^nd^ order diffraction spectrum that in turn starts straight after the 1^st^ order (see Fig. 5c).

After that, different periods obtained from the last experiment were selected for reaching a specific colour at the position d = 0 (see Fig. 1) in order to validate the hypothesis of this work. The same periods were used for processing a reference sample, in which the spatial periods were not changed as a function of the position d. The selected $\Lambda$ values are reported in Table 1.

**Table 1:** Utilised spatial periods for obtaining different structural colours when a constant (non-homogeneous strategy) and variable (homogenous strategy for increasing the homogeneity of structural colours) spatial periods over the sample surface are used (at a viewing angle of 22° and height of 50 cm, for d values ranging from 0 to 40 mm).

Colour	Spatial period (µm)	
	Non-homogenous strategy	Homogenous strategy
Blue	1.74	1.74–2.11
Light blue	1.83	1.83–2.21
Green	1.98	1.98–2.34
Yellow	2.16	2.16–2.62
Orange-yellow	2.20	2.20–2.66
Orange	2.23	2.23–2.70
Red	2.37	2.23–2.86

**Figure 6:**
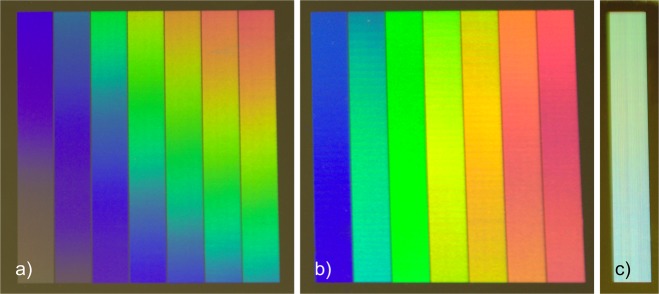
Stainless steel samples treated with (**a**) constant spatial periods; (**b**) variable spatial periods to increase the homogeneity of the structural colours and (**c**) with multiple variable spatial periods for mixing colours (following the strategy of Fig. 7).

A photograph of the treated reference stainless steel sample with constant spatial periods across the structured area is shown in Fig. 6a. As it can be seen, at the position d = 0 (upper part of the structured area), the target colours are in good agreement with the previous experimental results. However, due to the used constant spatial period to treat the sample, the characteristic rainbow colours could be observed across the processed areas (as explained before, see Fig. 1a).

Taking into consideration the length *d* of the treated area (d = 40 mm), we proceeded to calculate the needed spatial periods in order to obtain the homogeneous structural colours using Eqs 3 to 5. The calculated values are reported in Table 1. The results of the developed strategy are shown in Fig. 6b. As it can be seen in this case, the obtained colours are homogeneous and almost do not differ as a function of the position d.

Important to mention is the limitation in size of the images that can be produced, since larger images require larger variations of the spatial period. As it can be seen from Table 1, the red colour requires the largest spatial periods. Thus, taking into consideration that the largest period that can be produced with our optical setup is Λ = 4.59 µm, the largest size d of decorative elements that can be produced with this strategy can be calculated to d = 105 mm (for a viewing angle of 22° and height of 50 cm).

A disadvantage of the structural colours based on diffraction patterns, is the impossibility to obtain the white colour. The reason is due to the fact that the periodic structures decompose the white light as discussed before (see Eq. 1). However, it is known that white colour (or any other colour) can be obtained by mixing for instance red, green and blue light at certain intensities^38^. Thus, we also proceed to develop a strategy to produce homogenous white structural colour using DLIP.

**Figure 7:**
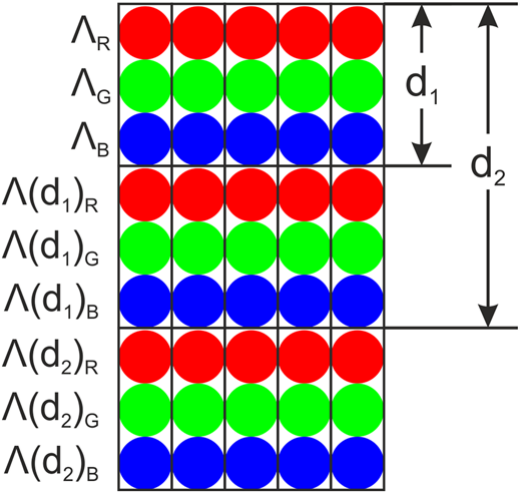
Developed strategy to obtain homogenous white structural colour, by mixing the diffracted blue, red and green wavelengths.

This strategy consisted on mixing periodic structures in order to obtain homogenous red, green and blue colours (also following the before reported strategy as well as the values reported in Table 1). Therefore, different rows for producing the above-mentioned colours were consecutively placed over the steel substrates as schematically shown in Fig. 7. After performing the calculations, an area of 5 mm by 40 mm was processed on the electro-polished stainless steel surface. The result can be observed in the photograph shown in Fig. 6c taken also at a viewing angle of 22° and height of 50 cm. As it can be seen in the image, the colour of the fabricated area is not completely white. This is because the intensity of each RGB component that is captured by the camera is different. As mentioned before, each colour component corresponds to a different wavelength, which requires a certain structure depth to reach the same diffraction efficiency (same colour intensity). However, as shown in Fig. 4, slight variations of the structure depth lead to significant changes in the diffraction intensity. Therefore, in order to improve the appearance of the white colour in the future, further calibrations are required, which consist of varying the structure depth for each individual colour and measuring the RGB intensity in the recorded picture so that the required ratio between the intensity of each colour components can be reached.

**Figure 8:**
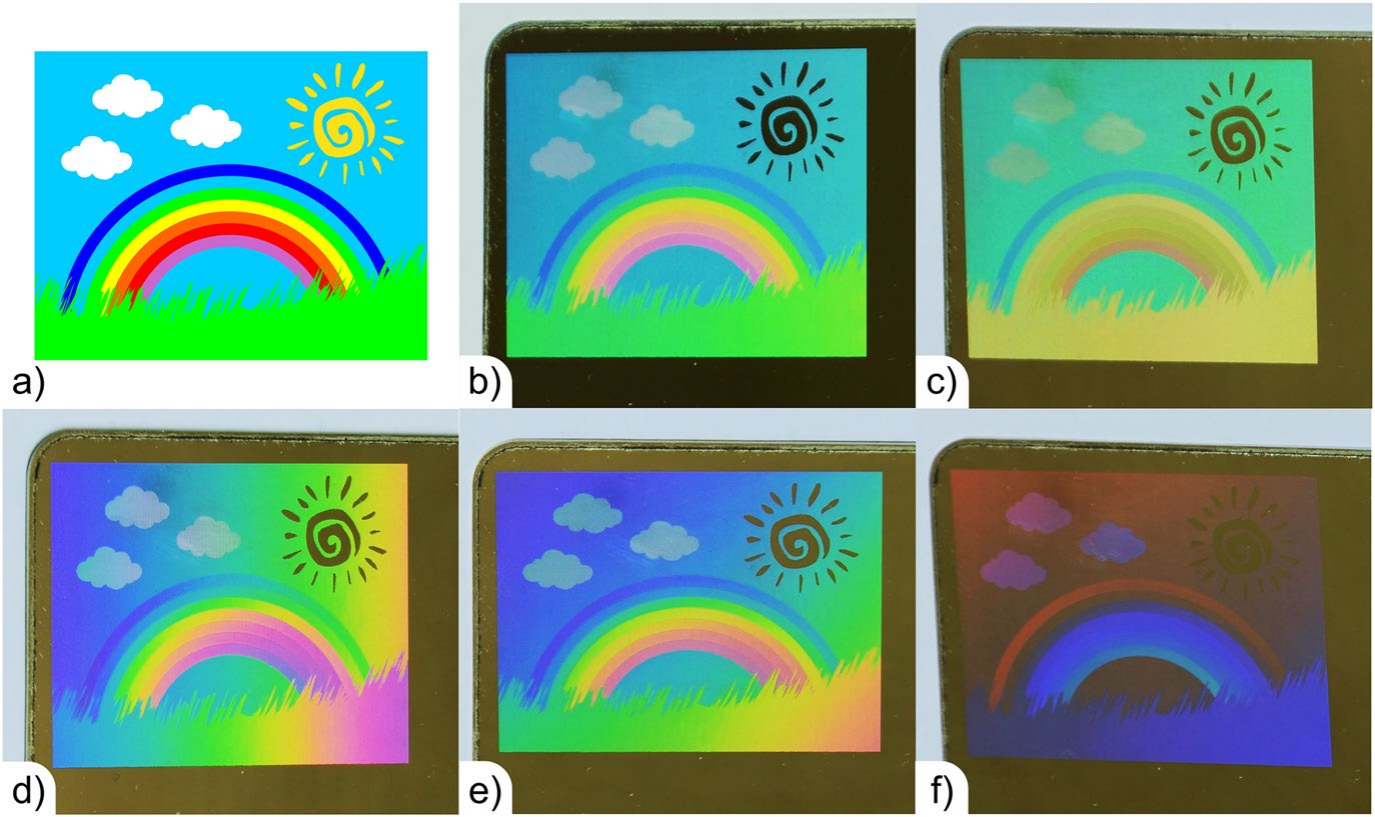
(**a**) Target “Rainbow” picture and observed structural colours under different viewing angles of (**b**) 22° (target angle) and (**c**) 25°; (**d**) at a viewing angle of 22°, but with the sample rotated by 180°, (**d**) 90° and (**e**) 45°.

Finally, we proceeded to produce a decorative image on stainless steel following the developed strategy. The target image (rainbow on a grass field, see Fig. 8a) has all 7 selected colours and additional white colour (clouds), which requires clusters of holographic pixels of green, red and blue colour. The sun in the image was left black, to show the possibility of producing this colour. In the last case, the area was not modified by the laser process. The size of the produced image was 40 mm by 30 mm and the spatial periods to fabricate each colour were calculated also with Eqs 3 to 5. The photographs of the fabricated image in Fig. 8b–f are rotated by 90°, which means that the top position (d = 0) is located at the left side of the image. As it can be observed in Fig. 8b, the obtained image is very similar to the target image at an observation angle of 22° and a height of 50 cm. Furthermore, the gradual vertical variation of the colours could be satisfactory eliminated for the target viewing angle. However, it has to be mentioned that these colours can be observed only for the selected viewing conditions. This means that under other observation positions, the colours will vary, but still their vertical variation can be avoided (see Fig. 8c).

In case when the sample is rotated around the surface normal, other effects arise. For example, when the sample is rotated by 180°, under the same observation angle (22°) and height (50 cm) the rainbow colour (gradual variation of the colour) will be observed in horizontal direction, since now the largest spatial periods are located at the position d = 0 mm (see Fig. 8d). A similar effect is also observed after rotating the sample 90°, but in this case, the rainbow affect is visible along the image diagonal (see Fig. 8e). When the sample is rotated by 45°, a significant drop in the intensity of the colours is observed, because the diffraction plane is misaligned with the observation angle (see Fig. 8f).

The manufacturing time for processing such image (40 mm × 30 mm) highly depends on both the used laser source and the beam positioning system. The used stages in this work permit a maximal speed of 200 mm/s. However, the dynamic properties of the particular stages are highly inertial which limited the fabrication speed down to 100 mm/s, resulting in a fabrication time of roughly 10 min. Nevertheless, the fabrication time can be highly reduced to few seconds when utilizing most powerful commercially available lasers (i.e. 100 W laser with 2 MHz frequency), as well as utilizing DLIP optics which have been already combined with galvanometer scanners, demonstrating processing speeds of 60 cm²/min^39^.

## Conclusions

In this work, a strategy to produce decorative elements with target structural colours was developed. This strategy consisted on producing periodic surface patterns with variable spatial period depending on the position at the substrate. Furthermore, using a mathematical model the needed spatial periods were calculated as a function of the target colour as well as the sample and viewer position.

The variable period patterns were fabricated on stainless substrates using DLIP and characterised by confocal microscopy and reflective spectrometry. A very good agreement between the observed and calculated structural colours for the defined observation conditions was achieved. In addition, a strategy for mixing colours and thus, for obtaining white colour (which cannot be produced directly using structural colours with periodic surface patterns), was developed. Finally, the developed model was successfully integrated into machine software, in order to automatically process images.

## Data Availability

The datasets generated and analysed during the current study are available from the corresponding author on reasonable request.
